# Association of Urinary Sodium Excretion and Left Ventricular Hypertrophy in People With Type 2 Diabetes Mellitus: A Cross-Sectional Study

**DOI:** 10.3389/fendo.2021.728493

**Published:** 2021-09-28

**Authors:** Jianfang Liu, Xiaoyu Yang, Peizhen Zhang, Dan Guo, Bingyan Xu, Chensihan Huang, Yaoming Xue, Huijie Zhang

**Affiliations:** ^1^Department of Endocrinology and metabolism, Nanfang Hospital, Southern Medical University, Guangzhou, China; ^2^Guangdong Provincial Key Laboratory of Shock and Microcirculation, Guangzhou, China; ^3^Department of Food Safety and Health Research Center, School of Public Health, Southern Medical University, Guangzhou, Guangdong, China

**Keywords:** urinary sodium excretion, left ventricular hypertrophy, cardiovascular disease, type 2 diabetes mellitus, metabolism

## Abstract

**Background:**

It has been well documented that left ventricular hypertrophy (LVH) is highly associated with the incidence of cardiovascular disease (CVD). Evidence indicated that high sodium intake was closely related with LVH in general population. However, information is not available regarding the association between urinary sodium excretion and LVH in patients with type 2 diabetes mellitus (T2DM). This study aimed to explore the association between urinary sodium excretion and LVH in patients with T2DM.

**Methods:**

This cross-sectional analysis included baseline data from 1,556 individuals with T2DM enrolled in the NanFang Prospective Diabetes Study (NFPDS). Urinary sodium excretion levels were measured from 24-hour urine samples of inpatients and morning fasting urine samples of outpatients. Left ventricular dimensions were assessed by echocardiography. The associations between urinary sodium excretion and the risks of cardiovascular events, LVH and left ventricular mass index (LVMI) were examined using linear regression analysis, logistic regression and restricted cubic splines (RCS).

**Results:**

Urinary sodium excretion levels were positively associated with cardiometabolic risk factors, including systolic blood pressure, body mass index, waist circumference and LVMI (All *P*<0.001). Odds ratios of the highest quartile of urinary sodium excretion compared with the lowest quartile were 1.80 (95% CI, 1.28-2.54; *P*=0.001) for LVH and 1.77 (95% CI, 1.06-2.94; *P*=0.028) for CVD, after adjusted for demographics, lifestyle risk factors and cardiovascular risk factors. Multivariable-adjusted RCS analysis of the association between urinary sodium excretion and LVMI showed a significant association (*P*=0.001) and lacked evidence of a nonlinear association (*P*=0.406).

**Conclusion:**

This study indicated that high urinary sodium excretion was independently associated with increased risk of LVH and CVD in patients with T2DM, suggesting that control of sodium intake may be valuable for the prevention of diabetic cardiovascular complications.

## Introduction

Cardiovascular disease (CVD) is the leading cause of death in patients with type 2 diabetes mellitus (T2DM) (1, 2). It affects 15% to 41% of middle-aged people with diabetes in western countries (3). In China, 14.6% of patients with T2DM have CVD (4). It has been well documented that left ventricular (LV) hypertrophy (LVH), an increase in LV mass (LVM), is an important predictor of congestive heart failure and cardiovascular mortality (5, 6). Consequently, investigation of the origins of LVH and CVD in patients with T2DM may have implications for developing preventive strategies for cardiovascular complications in diabetes.

It has been proposed that increased sodium intake has a direct relationship with the risk of LVH (7–10). The proposed pathogenetic mechanisms linking sodium intake to LVH include elevated activity of the renin angiotensin system (11), increased reactivity of sympathetic nervous system (12) and an increase in the cardiac volume load (13, 14). Longitudinal studies have indicated that urinary sodium excretion was significant associated with the incidence of LVH in both normotensive (15, 16) and hypertensive individuals (9, 10, 17–19). However, the relationship between urinary sodium excretion levels and LVH in patients with T2DM remains largely unclear. The aim of current study was to explore the association between urinary sodium excretion levels and LVH in patients with T2DM.

## Materials and Methods

### Study Participants

This cross-sectional study was derived from the NanFang Prospective Diabetes Study (NFPDS), a prospective cohort study designed to explore the associations of possible risk factors with T2DM complications in Chinese population. Individuals over aged 20 years old with T2DM were recruited from the Nanfang Hospital of Southern Medical University, Guangzhou, China. Individuals with any of the following conditions were excluded: 1) NYHA class III or IV congestive heart failure; 2) treatment with dialysis; 3) severe systemic infections; 4) females who were pregnant, or planning to become pregnant; 5) lack of signed informed consent. The study comprised 1,556 individuals with valid urine collections and LV dimension measurements were included from January 2018 to December 2020. All subjects completed a uniform questionnaire including social-demographic status, lifestyle habits (i.e., smoking status, alcohol consumption) and medical history. Hypertension was defined as mean blood pressure (BP) of 140/90 mmHg or greater or self-reported use of antihypertensive medication. Hyperlipidemia was defined as total cholesterol (TC) ≥ 6.22mmol/L, or low-density lipoprotein cholesterol (LDL-c) ≥ 4.14 mmol/L, or triglycerides (TG) ≥ 2.26 mmol/L. The presence of CVD was defined as a clinical history of coronary heart disease, myocardial infarction, unstable angina requiring hospitalization, heart failure, stroke and peripheral vascular disease.

Written informed consent was obtained from each individual. The study protocol was approved by the Institutional Review Board of Nanfang Hospital of Southern Medical University. The methods were carried out in accordance with the approved guidelines.

### Clinical and Biochemical Measurements

Anthropometric measurements included height, weight, waist circumference and BP. Body mass index (BMI) was calculated as weight in kilograms divided by the square of the height in meters. Waist circumference was measured at the level of 1cm above the umbilicus. Three measurements were obtained with a non-stretchable tape, and the mean value was used for analysis. BP was assessed in triplicate using an electronic sphygmomanometer (OMRON Company).

Blood samples of all individuals were collected after 12-hour fasting and tested at the laboratory of Nanfang hospital. Fasting plasma glucose concentrations were measured using the hexokinase method. Glycated hemoglobin (HbA1c) was measured by high performance liquid chromatography. TG, TC, and LDL-c were measured by enzymatic methods using a fully automated biochemical analyzer.

For inpatients, 24-hour urine specimens were measured for urinary sodium excretion, which is generally considered the most reliable estimate of sodium intake (20). A fasting, morning spot urine sample was obtained from outpatients. For outpatients, the 24-hour urinary sodium excretion was estimated using the Kawasaki formula (21, 22). 24-hour and spot urinary sodium concentrations were measured using ion selective electrode method.

### Echocardiography

LV dimensions were assessed by 2-dimensional guided M-mode echocardiography with 2.25- and 3.5-MHz transducers according to American Society of Echocardiography recommendations (23). The echocardiography was performed in all participants by trained radiologist in ultrasound, who was blinded to the individuals’ specific medical information/health status. LVM was calculate using the Penn-cube method, a necropsy-validated formula: LVM = 1.04 [(interventricular septal thickness + LV internal dimension + posterior wall thickness)^3^ - LV internal dimension^3^] - 13.6 (24). To reduce the confounding effects of body size, LVM was indexed for body height (m^2.7^) as LV mass index (LVMI). The presence of left ventricular hypertrophy (LVH) was defined LVMI >46.7 g/m^2.7^ in women and LVMI >49.2 g/m^2.7^ in men (25). Relative wall thickness was calculated as interventricular septal thickness plus posterior wall thickness divided by LV end-diastolic diameter.

### Statistical Analysis

Continuous variables are presented as means ± standard deviation (SD) or median (interquartile range), and categorical variables are presents as frequencies and percentages. Data that were not normally distributed were logarithmically transformed before analysis. Baseline characteristics of study participants were compared across quartiles of urinary sodium excretion using general linear models (GLM) for continuous variables and χ^2^ -test for categorical variables. Multivariable linear regression analyses were used to investigate the association of the following variables with urinary sodium excretion: BMI, waist circumference, systolic BP (SBP), diastolic BP (DBP), duration of diabetes, HbA1c, fasting glucose, TC, TG, LDL-c and LVMI. Multivariable logistic regression models were used to examine the association of urinary sodium excretion levels with risks of LVH and CVD. Forest plot was used to present the relationship between urinary sodium excretion levels and LVH in different subgroups. Interaction tests were done to assess whether the association between urinary sodium excretion level and LVH differed in subgroups. The relationship between urinary sodium excretion and LVMI was examined with restricted cubic splines (RCS) (26). Analyses were multivariable-adjusted and used 5 knots (located at the 5th, 25th, 50th, 75th, and 95th percentiles). The World Health Organization (WHO) has recommended that sodium intake should be below 2g/day, patients with 2g/day of urinary sodium excretion were chosen as the reference group (27). Statistical analyses were performed with SAS version 9.4 (SAS Institute Inc) and R version 4.1.0. Two-sided values of *P* < 0.05 were considered statistically significant.

## Results

**Table 1** summarizes demographic and clinical characteristics of individuals categorized by quartiles of urinary sodium excretion levels. A total of 1,556 individuals (mean age, 55 years; 64% males) with T2DM were included in this study. Overall, the average duration of diabetes was 8 years and the mean ± SD of the estimated 24-hour urinary sodium excretion was 3.29 ± 1.43 g/day. Males had higher urinary sodium excretion than females. Individuals with higher urinary sodium excretion had longer duration of diabetes and higher proportion of smoking and take antidiabetic medications. Compared to individuals in the lowest quartile of urinary sodium excretion, those in the highest quartile had higher levels of BMI and waist circumference, and lower levels of HbA1c, TC, LDL-c. Of note, the level of LVMI and the proportion of LVH was significantly higher in individuals with highest levels of urinary sodium excretion than those with lowest values after adjusted for age and gender.

**Table 1:** Characteristics of individuals categorized by quartile of urinary sodium excretion.

Variables	Estimated 24-hour urinary sodium excretion level				*P*-value
	Quartile 1 (n = 389)	Quartile 2 (n = 389)	Quartile 3 (n = 389)	Quartile 4 (n = 389)	
Urinary sodium (g/day)	1.72 ± 0.44	2.73 ± 0.25	3.52 ± 0.26	5.19 ± 1.19	–
Outpatient (n, %)	64 (17)	136 (35)	170 (44)	176 (45)	<0.001
Age (years)	55 ± 12	56 ± 11	56 ± 11	54 ± 11	0.137
Gender (Male n, %)	217 (56)	237 (61)	257 (66)	281 (72)	<0.001
Smoking (n, %)	160 (41)	162 (42)	193 (50)	199 (51)	0.004
Alcohol use (n, %)	118 (30)	130 (34)	152 (39)	130 (34)	0.066
Duration of diabetes (years)	4 (1-11)	7 (2-13)^‡^	8 (3-13)^‡^	7 (3-13) ^‡^	<0.001
BMI (kg/m^2^)	24.0 ± 3.6	24.3 ± 3.2	24.6 ± 3.3^†^	25.5 ± 3.7^‡^	<0.001
Waist circumference (cm)	86.6 ± 9.7	87.6 ± 8.9	88.8 ± 8.9^‡^	90.6 ± 9.9^‡^	<0.001
SBP (mmHg)	127 ± 18	127 ± 19	128 ± 18	129 ± 18^†^	0.126
DBP (mmHg)	77 ± 11	77 ± 10	76 ± 11	78 ± 11	0.053
Hypertension (n, %)	155 (40)	136 (35)	130 (33)	133 (34)	0.234
Hyperlipidemia (n, %)	233 (60)	232 (60)	235 (60)	219 (56)	0.640
CVD (n, %)	38 (9.8)	54 (14)	46 (12)	57 (15)^†^	0.163
Antidiabetic medication (n, %)	245 (63)	286 (74)	313 (81)	311 (80)	<0.001
RAS blocking agents (n, %)	66 (17)	57 (15)	72 (19)	58 (15)	0.416
Diuretics (n, %)	18 (4.6)	8 (2.1)	8 (2.1)	11 (2.8)	0.106
Statin (n, %)	50 (13)	53 (14)	58 (15)	54 (14)	0.872
Aspirin (n, %)	36 (9.3)	29 (7.5)	38 (9.8)	38 (9.8)	0.635
HbA1c (%)	10.1 ± 2.7	9.4 ± 2.5^‡^	8.9 ± 2.4^‡^	8.9 ± 2.2^‡^	<0.001
Fasting glucose (mmol/L)	7.71 (5.56-10.37)	7.42 (5.59-10.33)	7.43 (5.55-9.85)	7.44 (5.78-9.83)	0.236
TG (mmol/L)	1.38 (0.96-2.32)	1.52 (1.03-2.42)	1.48 (1.03-2.36)	1.58 (1.05-2.56)	0.485
TC (mmol/L)	5.06 ± 1.51	5.14 ± 1.30	4.88 ± 1.31	4.97 ± 1.29	0.044
LDL-c (mmol/L)	3.23 ± 0.99	3.32 ± 0.97	3.14 ± 0.96	3.15 ± 0.84	0.025
LVM (g)	155.0 ± 40.7	163.5 ± 42.7^†^	167.1 ± 44.7^‡^	171.2 ± 47.9^‡^	<0.001*
LVMI (g/m^2.7^)	42.6 ± 11.4	44.7 ± 11.7^†^	45.2 ± 12.3^‡^	45.1 ± 13.0^‡^	0.001*
RWT	0.50 ± 0.09	0.50 ± 0.09	0.50 ± 0.09	0.50 ± 0.09	0.986
LVH (n, %)	111 (29)	137 (35) ^†^	128 (33)	137 (35)^‡^	0.004*

BMI, body mass index; SBP, systolic blood pressure; DBP, diastolic blood pressure; CVD, cardiovascular disease; RAS, renin-angiotensin system; HbA1c, glycated hemoglobin; TG, triglycerides; TC, total cholesterol; LDL-c, low-density lipoprotein cholesterol; LVM, left ventricular mass; LVMI, left ventricular mass index; RWT, relative wall thickness; LVH, left ventricular hypertrophy.

^†^P < 0.05 compared with Quartile 1 of urinary sodium excretion.

^‡^P < 0.01 compared with Quartile 1 of urinary sodium excretion.

*Adjusted for age and gender.

Clinical characteristics by gender and quartile of urinary sodium excretion levels are shown in **Table 2**. Both males and females with the highest quartile had higher levels of BMI and waist circumference, and lower levels of HbA1c, TC and LDL-c. Compared with the lowest quartiles of urinary sodium excretion, the level of LVMI and the proportion of LVH were significantly increased in the higher quartiles in males after adjusted for age.

**Table 2:** Characteristics of individuals categorized by quartile of urinary sodium excretion and gender.

Variables	Males (n = 992)				*P*-value	Females (n = 564)				*P*-value
	Estimated 24-hour urinary sodium excretion level					Estimated 24-hour urinary sodium excretion level				
	Quartile 1 (n = 248)	Quartile 2 (n = 248)	Quartile 3 (n = 248)	Quartile 4 (n = 248)		Quartile 1 (n = 141)	Quartile 2 (n = 141)	Quartile 3 (n = 141)	Quartile 4 (n = 141)	
Urinary sodium (g/day)	1.83 ± 0.47	2.86 ± 0.25	3.67 ± 0.29	5.40 ± 1.26	–	1.57 ± 0.36	2.53 ± 0.24	3.29 ± 0.22	4.77 ± 1.03	–
Outpatient (n, %)	51 (21)	88 (36)	115 (46)	100 (40)	<0.001	18 (13)	45 (32)	58 (41)	71 (50)	<0.001
Age (years)	51 ± 11	54 ± 11^‡^	54 ± 11^†^	53 ± 11	0.020	59 ± 11	59 ± 9	59 ± 10	57 ± 9	0.385
Smoking (n, %)	178 (72)	168 (68)	181 (73)	170 (69)	0.484	4 (2.8)	0 (0.0)	7 (5.0)	6 (4.3)	0.070
Alcohol use (n, %)	122 (49)	122 (49)	134 (54)	109 (44)	0.154	8 (5.7)	13 (9.2)	12 (8.6)	10 (7.1)	0.680
Duration of diabetes (years)	3 (1-8)	6 (2-12)^‡^	8 (2-12)^‡^	7 (3-13)^‡^	<0.001	6 (2-12)	9 (3-14)	9 (4-13)	8 (3-15)	0.521
BMI (kg/m^2^)	23.9 ± 3.7	24.2 ± 2.9	24.7 ± 3.6^†^	25.6 ± 3.6^‡^	<0.001	23.6 ± 3.5	24.9 ± 3.2^‡^	24.6 ± 3.2^†^	25.1 ± 3.9^‡^	0.002
Waist circumference (cm)	87.5 ± 10.0	88.0 ± 8.4	89.6 ± 9.6	91.6 ± 9.6	<0.001	85.1 ± 9.2	87.4 ± 8.8	87.6 ± 8.5	87.9 ± 10.1	0.044
SBP (mmHg)	123 ± 18	126 ± 19	127 ± 18^†^	129 ± 16^‡^	0.004	129 ± 18	131 ± 18	129 ± 18	131 ± 22	0.790
DBP (mmHg)	78 ± 12	77 ± 10	78 ± 11	80 ± 10^†^	0.020	76 ± 11	77 ± 10	74 ± 10	76 ± 12	0.105
Hypertension (n, %)	82 (33)	68 (27)	79 (32)	83 (27)	0.448	67 (48)	63 (45)	59 (42)	53 (38)	0.377
Hyperlipidemia (n, %)	150 (61)	147 (59)	143 (58)	145 (59)	0.931	80 (57)	87 (62)	90 (64)	77 (55)	0.362
CVD (n, %)	19 (7.7)	26 (11)	32 (13)	35 (14)^†^	0.110	20 (14)	23 (16)	23 (16)	17 (12)	0.706
Antidiabetic medication (n, %)	140 (57)	168 (68)	197 (79)	194 (78)	<0.001	101 (72)	115 (82)	123 (87)	117 (83)	0.008
RAS blocking agents (n, %)	33 (13)	30 (12)	45 (18)	37 (15)	0.251	31 (22)	28 (20)	30 (21)	19 (14)	0.249
Diuretics (n, %)	8 (3.2)	5 (2.0)	5 (2.0)	6 (2.4)	0.795	8 (5.7)	5 (3.6)	4 (2.8)	4 (2.8)	0.547
Statin (n, %)	22 (8.9)	23 (9.3)	41 (17)	33 (13)	0.026	29 (21)	21 (15)	31 (22)	15 (11)	0.041
Aspirin (n, %)	16 (6.5)	20 (8.1)	22 (8.9)	24 (9.7)	0.602	19 (14)	7 (5.0)	20 (14)	13 (9.2)	0.041
HbA1c (%)	10.5 ± 2.7	9.4 ± 2.7^‡^	8.8 ± 2.3^‡^	9.0 ± 2.4^‡^	<0.001	9.7 ± 2.6	9.1 ± 2.4	8.9 ± 2.2^‡^	8.5 ± 2.1^‡^	<0.001
Fasting glucose (mmol/L)	8.23 (5.90-11.10)	7.67 (5.72-10.95)	7.18 (5.58-9.44)^‡^	7.40 (5.65-9.82)^†^	0.002	7.03 (5.19-9.79)	7.14 (5.51-9.20)	7.35 (5.61-9.94)	7.69 (5.74-10.31)	0.864
TG (mmol/L)	1.39 (0.92-2.58)	1.49 (1.03-2.53)	1.44 (0.97-2.39)	1.61 (1.09-2.90)	0.342	1.30 (0.97-1.99)	1.63 (1.09-2.30)	1.51 (1.19-2.19)	1.54 (1.02-2.15)	0.254
TC (mmol/L)	5.16 ± 1.66	5.08 ± 1.22	4.82 ± 1.43^‡^	4.86 ± 1.31^†^	0.019	4.78 ± 1.16	5.32 ± 1.32^‡^	4.99 ± 1.26	5.17 ± 1.12^‡^	0.002
LDL-c (mmol/L)	3.28 ± 1.02	3.30 ± 0.91	3.09 ± 0.99^†^	3.09 ± 0.81^†^	0.009	3.05 ± 094	3.44 ± 0.99^‡^	3.20 ± 0.94	3.28 ± 0.90^†^	0.005
LVM (g)	159.7 ± 44.2	166.7 ± 46.1	177.6 ± 46.1^‡^	176.5 ± 47.8^‡^	<0.001*	148.2 ± 33.8	155.2 ± 35.5	153.4 ± 39.5	158.5 ± 43.1^†^	0.066*
LVMI (g/m^2.7^)	40.1 ± 11.6	42.1 ± 11.6	44.8 ± 12.1^‡^	44.1 ± 12.6^‡^	<0.001*	45.5 ± 10.8	47.9 ± 10.5^‡^	47.2 ± 12.0	48.1 ± 13.4^†^	0.094*
RWT	0.48 ± 0.09	0.49 ± 0.08	0.50 ± 0.09^‡^	0.50 ± 0.08^‡^	0.133	0.51 ± 0.10	0.51 ± 0.09	0.52 ± 0.10	0.50 ± 0.10	0.330
LVH (n, %)	46 (19)	57 (23)	69 (28)^†^	72 (29)^†^	0.005*	55 (39)	79 (56)	62 (44)	73 (52)	0.073*

BMI, body mass index; SBP, systolic blood pressure; DBP, diastolic blood pressure; CVD, cardiovascular disease; RAS, renin-angiotensin system; HbA1c, glycated hemoglobin; TG, triglycerides; TC, total cholesterol; LDL-c, low-density lipoprotein cholesterol; LVM, left ventricular mass; LVMI, left ventricular mass index; RWT, relative wall thickness; LVH, left ventricular hypertrophy.

^†^P < 0.05 compared with Quartile 1 of urinary sodium excretion.

^‡^P < 0.01 compared with Quartile 1 of urinary sodium excretion.

*Adjusted for age.

Results of linear regression analysis of urinary sodium excretion on cardiovascular risk factors are shown in **Table 3**. Urinary sodium excretion levels were significantly correlated with BMI, SBP, DBP, waist circumference, duration of diabetes, LVMI and HbA1c after adjusting age, gender, smoking, alcohol consumption and use of renin-angiotensin system (RAS) blocking agents, diuretics, statin and antidiabetic medication (all *P*<0.05). There were not significant associations between urinary sodium excretion with TG, TC and fasting glucose after adjusted for demographics, lifestyle risk factors and current medication use.

**Table 3:** Clinical correlation of urinary sodium excretion levels with clinical and biochemical variables.

Variables	Regression coefficient β	Standard error	*P*-value	Multiple adjusted *P*-value^§^
BMI (kg/m^2^)	0.549	0.088	<0.001	<0.001
Waist circumference (cm)	1.517	0.243	<0.001	<0.001
SBP (mmHg)	1.737	0.464	<0.001	<0.001
DBP (mmHg)	0.811	0.275	0.003	0.023
Duration of diabetes (year)	0.689	0.175	<0.001	0.002
HbA1c (%)	-0.420	0.063	<0.001	<0.001
Fasting glucose (mmol/L)	0.002	0.011	0.894	0.361
TG (mmol/L)	0.021	0.017	0.216	0.429
TC (mmol/L)	-0.072	0.034	0.037	0.523
LDL-c (mmol/L)	-0.043	0.024	0.074	0.050
LVMI (g/m^2.7^)	0.877	0.308	0.005	<0.001

BMI, body mass index; SBP, systolic blood pressure; DBP, diastolic blood pressure; HbA1c, glycated hemoglobin; TG, triglycerides; TC, total cholesterol; LDL-c, low-density lipoprotein cholesterol; LVMI, left ventricular mass index; RAS, renin-angiotensin system.

^§^Adjusted for age, gender, smoking, alcohol consumption, RAS blocking agents, diuretics, statin antidiabetic medication and serum creatinine.

The odds ratios (ORs) with 95% confidence interval (CI) for LVH and CVD according to changes in urinary sodium excretion levels are showed in **Table 4**. Compared with individuals in quartile 1, the risk of LVH was significantly increased in quartile 2 [OR (95% CI), 1.59 (1.14-2.21; *P*=0.007], quartile 3 [OR (95% CI), 1.52 (1.08-2.11); *P*=0.017] and quartile 4 [OR (95% CI), 1.92 (1.37-2.68); *P*<0.001] after adjusted for age, gender, smoking status, alcohol consumption, history of hypertension and hyperlipidemia, and use of antihypertension and antihyperlipidemic medication. These relationships also remained significant when further adjusting for HbA1c and use of antidiabetic medication. Furthermore, individuals in the highest quartile had significantly higher risks of CVD than those in the lowest quartile, even after adjusting for demographics, lifestyle risk factors and cardiovascular risk factors (all *P*<0.05).

**Table 4:** Odds ratios (ORs) of LVH and CVD according to urinary sodium excretion levels.

Variables	LVH			CVD		
	OR	95% CI	*P*-value	OR	95% CI	*P*-value
Crude model						
urinary sodium (g/day)						
(Quartile 2 *vs.* Quartile 1)	1.36	1.01-1.84	0.046	1.49	0.96-2.31	0.077
(Quartile 3 *vs.* Quartile 1)	1.23	0.91-1.67	0.187	1.24	0.79-1.95	0.357
(Quartile 4 *vs.* Quartile 1)	1.36	1.01-1.84	0.046	1.59	1.02-2.45	0.039
Model 1						
urinary sodium (g/day)						
(Quartile 2 *vs.* Quartile 1)	1.43	1.04-1.97	0.029	1.49	0.94-2.36	0.088
(Quartile 3 *vs.* Quartile 1)	1.33	0.96-1.85	0.082	1.18	0.73-1.90	0.504
(Quartile 4 *vs.* Quartile 1)	1.74	1.26-2.41	0.001	1.79	1.13-2.84	0.014
Model 2						
urinary sodium (g/day)						
(Quartile 2 *vs.* Quartile 1)	1.59	1.14-2.21	0.007	1.65	1.01-2.69	0.047
(Quartile 3 *vs.* Quartile 1)	1.51	1.08-2.11	0.017	1.15	0.69-1.93	0.595
(Quartile 4 *vs.* Quartile 1)	1.92	1.37-2.68	<0.001	1.93	1.17-3.17	0.010
Model 3						
urinary sodium (g/day)						
(Quartile 2 *vs.* Quartile 1)	1.53	1.09-2.14	0.013	1.57	0.96-2.58	0.073
(Quartile 3 *vs.* Quartile 1)	1.41	1.00-2.00	0.048	1.07	0.64-1.81	0.794
(Quartile 4 *vs.* Quartile 1)	1.80	1.28-2.54	0.001	1.77	1.06-2.94	0.028

LVH, left ventricular hypertrophy; CVD, cardiovascular disease; DBP, diastolic blood pressure; RAS, renin-angiotensin system; HbA1c, glycated hemoglobin.

Crude model: without adjustment.

Model 1: adjusted for age, gender, smoking and alcohol consumption.

Model 2: adjusted for model 1+ hypertension, RAS blocking agents, diuretics, hyperlipidemia and statin.

Model 3: adjusted for model 2+ HbA1c and antidiabetic medication.

**Figure 1:**
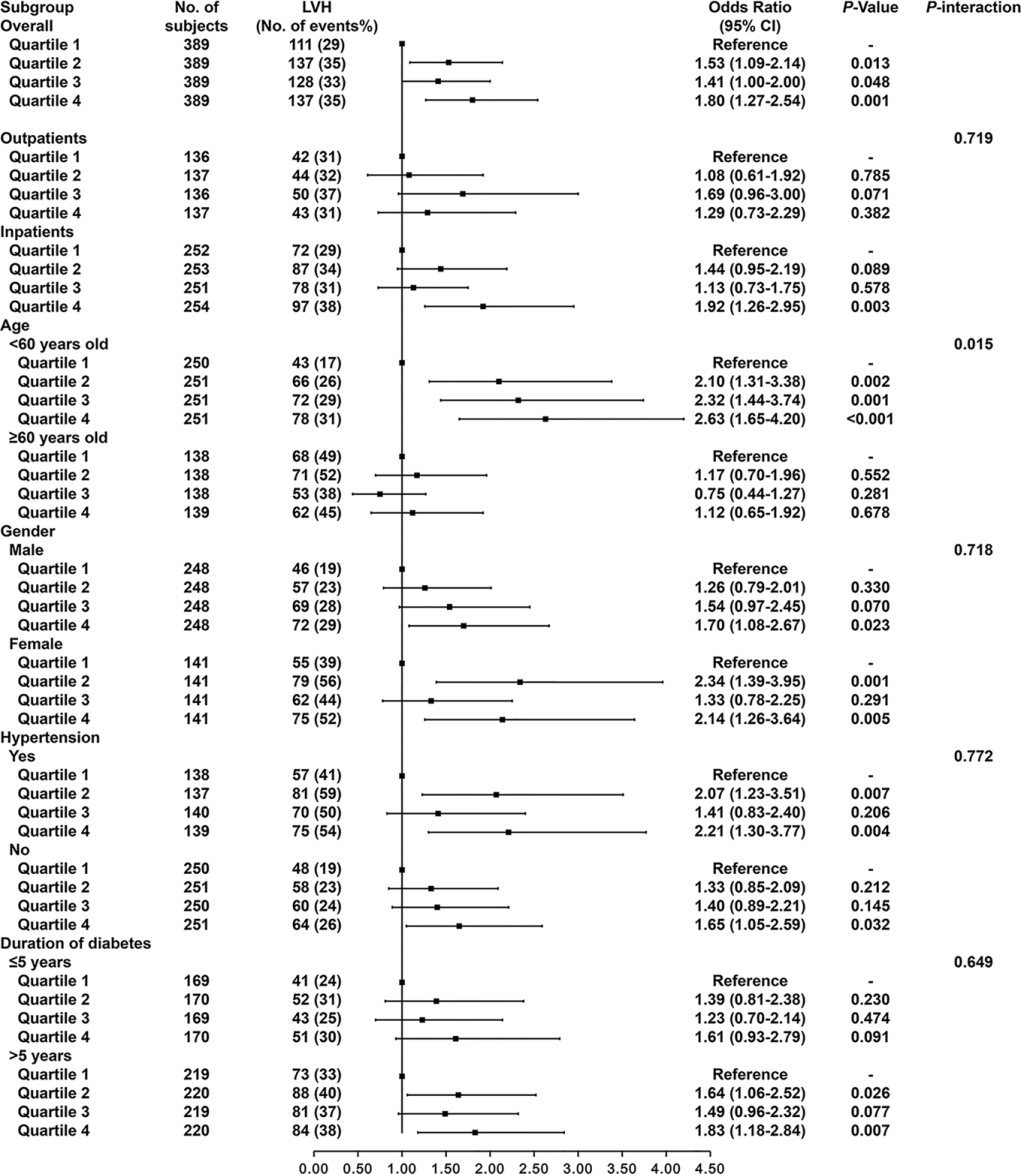
Odds ratios of LVH according to urinary sodium excretion levels in different subgroups. Adjusted for age, gender, smoking, alcohol consumption, hypertension, use of RAS blocking agents, use of diuretics, history of hyperlipidemia, use of statin, HbA1c and use of antidiabetic medication.

In addition, the subgroup analysis was performed to explore the association between LVH and urinary sodium excretion levels among different populations, according to the following variables: outpatients/inpatients, age (<60 years/≥60 years), gender (male/female), hypertension (yes/no), duration of diabetes (≤5 years/>5 years) (**Figure 1**). The association of urinary sodium excretion with the LVH was significant in the individuals aged under 60 years old (*P* for interaction=0.015). However, there were no significant differences in the associations between urinary sodium excretion and LVH among another subgroups (all *P* for interaction>0.05).

**Figure 2:**
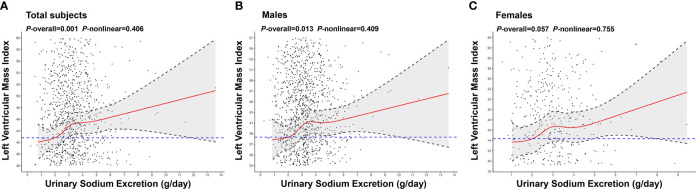
Association of urinary sodium excretion with LVMI. The restricted cubic spline (RCS) regression was used to analyze the relationship of urinary sodium excretion(g/day) with left ventricular mass index (LVMI) after adjusting for age, gender, smoking, alcohol consumption, hypertension, use of RAS blocking agents, use of diuretics, history of hyperlipidemia, use of statin, HbA1c and use of antidiabetic medication in total individuals **(A)**, males **(B)**, females **(C)**. Urinary sodium excretion was coded using an RCS function with five knots located at the 5th, 25th, 50th, 75th, 95th percentiles of the distribution of urinary sodium excretion. Y-axis represents the predicted value of LVMI. Individuals with 2g/day of urinary sodium excretion was the reference standard, indicated by the blue dashed lines. Black dots: “observed” data points. Black dashed lines: 95 percent confidence interval.

Multivariable-adjusted RCS analyses demonstrated that urinary sodium excretion was significantly associated with LVMI in overall patients (*P*=0.001) and males (*P*=0.013). However, the non-linear associations of urinary sodium excretion and LVMI were not significant (all *P*>0.05) (**Figure 2**). No significant relationship between urinary sodium excretion and LVMI was found in females (*P*=0.057).

## Discussion

It has been documented that dietary sodium intake plays a role in modulating myocardial mass and cardiac structure (28, 29). Evidence from experimental studies indicated that dietary sodium loading promotes cellular hypertrophy in both arterial smooth muscle and cardiac myocytes, as well as interstitial fibrosis in the left ventricle and myocardial arteries (30, 31). Proposed mechanisms including fluid homeostasis, neurohormonal regulation and several novel pathways (29). In the present study, our data indicated that diabetic patients with high urinary sodium excretion had higher level of LVMI. Additionally, we found that a J-shaped association between urinary sodium excretion and LVMI, instead of the direct linear correlation. Urinary sodium excretion was positively associated with cardiometabolic risk factors, including SBP, BMI and waist circumference. Of note, we provided the novel evidence that diabetic patients with higher urinary sodium excretion had a significantly increased risk of LVH and CVD independent of demographic factors, lifestyle risk factors and cardiovascular risk factors. These findings suggest that moderate sodium reduction among patients with T2DM may lower the risks of LVH and CVD.

It has been established that sodium intake influences the cardiovascular system profoundly in general population (14–18). Several studies have reported that increased urinary sodium excretion was significantly associated with greater LVM in healthy young adults and individuals with hypertension (8, 15, 19). In contrast, recent study indicated that dietary sodium restriction was associated with the decreased of LVM in hypertensive patients, even in those with proper BP control (32).However, information regarding the association of urinary sodium excretion with increased LVMI in individuals with T2DM is limited. In the present study, we found a positively association between urinary sodium excretion and LVMI in patients with T2DM. Of note, our data demonstrated that higher urinary sodium excretion concentrations were independently associated with increased risk of LVH in patients with T2DM. Our findings were consistent with prior studies in patients with essential hypertension (9, 10, 14). Additionally, several epidemiological studies reported that high sodium intake is strongly and independently associated with an increased risk of CVD and all-cause mortality (33, 34). These studies are congruent with our findings that high urinary sodium excretion was significantly associated with an increased risk of CVD in patients with T2DM. These findings have implications for monitoring sodium intake among T2DM patients.

In addition, several studies reported that the association between sodium consumption and CVD or mortality is J-shaped in general population (35–37). Evidence indicated that individuals with estimated sodium intake between 3 g/day and 6 g/day was associated with a lower risk of cardiovascular morbidity and mortality than those either with a higher or lower estimated level of intake (35, 36). In the present study, a possible J-shaped association was found between sodium excretion and LVMI in patients with T2DM. In addition, our data indicated that individuals with 2 g/day of urinary sodium excretion were associated with lower levels of LVMI, supporting that the sodium intake limitation of 2 g/day recommended by WHO and American Diabetes Association may also be suitable for patients with T2DM (27, 38).

Extensive observations have indicated that obesity and hypertension are the most important determinants of LVH in the general population (39–41). Elevated BP plays a driving role in the development of LVH through chronic hemodynamic overload and increased central pressure (42). Our data demonstrated that urinary sodium excretion was significantly and positively correlated with SBP after adjusted for demographics and lifestyle risk factors. Furthermore, our data showed that the association of sodium intake with LVH remained significant after adjusted for the history of hypertension. These data indicated that other mechanisms may play a role in the effect of dietary sodium on LVH in individuals with T2DM, which was consistent with previous findings of a positive association between dietary sodium intake and CVD independent of blood pressure (43, 44). Some possible mechanisms independent of blood pressure include circulating fluid volume (13), insulin resistance (45, 46) and obesity (41). Evidence from epidemiological studies have indicated that adiposity is one of the major predictors of LVH (41). Cross-sectional studies have demonstrated that high sodium intake is independently associated with elevated risk of obesity and central obesity (47, 48). In consist, our data also indicated that urinary sodium excretion was positively correlated with BMI and waist circumference. These results suggest that high sodium intake was significant associated with CVD risks, and may be an important risk factor for LVH and CVD risks among individuals with T2DM.

To our knowledge, this is the first analysis of the association between urinary sodium excretion levels and LVH in patients with T2DM, which can provide novel evidence for cardiovascular risk management in patients with T2DM. However, several limitations of this study must be considered. First, this was a cross-sectional study. Thus, it was not possible to determine a causal relationship between urinary sodium excretion and the development of LVH. Second, we used spot urine samples instead of 24-hour urine collection for the estimation of sodium excretion in outpatients. However, the Kawasaki formula is recognized as one of the most valid and least biased methods of estimating 24-h urinary sodium excretion (49). Third, it was not possible to determine clinical outcomes among individuals with urinary sodium excretion less than 2g/day due to the very small sample sizes among these subgroups.

## Conclusions

In summary, our study provides the clinical evidence revealing that high urinary sodium excretion levels were independently associated with increased risks of LVH and CVD in patients with T2DM. These findings suggests that it is critical to control sodium intake for the prevention of CVD in patients with T2DM. Further prospective studies need to determine the associations of urinary sodium excretion with cardiovascular complications in patients with T2DM.

## Data Availability Statement

The raw data supporting the conclusions of this article will be made available by the authors, without undue reservation.

## Ethics Statement

The studies involving human participants were reviewed and approved by the Institutional Review Board of Nanfang Hospital of Southern Medical University. The patients/participants provided their written informed consent to participate in this study.

## Author Contributions

JL, YX, and HZ contributed to conception and design of the study. YX and HZ supervised the study. DG, BX, and CH organized the database. JL, XY, and PZ performed the statistical analysis. JL wrote the first draft of the manuscript. JL, XY, PZ, and DG wrote sections of the manuscript. All authors contributed to the article and approved the submitted version.

## Funding

This study was supported by the National Key Research and Development Project (No.2018YFA0800404), Natural Science Foundation and Key-Area Research and Development Program of Guangdong Province (Nos.2018B030311031 and 2019B020227004), the National Natural Science Foundation of China (No.81970736), and Key-Area Clinical Research Program of Southern Medical University (No.LC2019ZD010). HZ was partially supported by Outstanding Youths Development Scheme of Nanfang Hospital, Southern Medical University (No. 2017J005).

## Conflict of Interest

The authors declare that the research was conducted in the absence of any commercial or financial relationships that could be construed as a potential conflict of interest.

## Publisher’s Note

All claims expressed in this article are solely those of the authors and do not necessarily represent those of their affiliated organizations, or those of the publisher, the editors and the reviewers. Any product that may be evaluated in this article, or claim that may be made by its manufacturer, is not guaranteed or endorsed by the publisher.
